# Circulating Tumor Cells Enhance Prognostic Stratification Beyond ER Assessment by Biopsy or FES-PET in Endocrine-Treated Metastatic Breast Cancer

**DOI:** 10.3390/diagnostics16081197

**Published:** 2026-04-17

**Authors:** Bertha Eisses, Lindsay Angus, Evelien J. M. Kuip, C. Willemien Menke-van der Houven van Oordt, Bert van der Vegt, Andor W. J. M. Glaudemans, Adrienne H. Brouwers, Daniela E. Oprea-Lager, Wim J. G. Oyen, Jasper Emmering, Anieta M. Sieuwerts, Agnes Jager, Jaco Kraan, John W. M. Martens, Elisabeth G. E. de Vries, Stefan Sleijfer, Carolina P. Schröder

**Affiliations:** 1 Department of Medical Oncology, University Medical Center Groningen, University of Groningen, 9713 GZ Groningen, The Netherlands; b.eisses@umcg.nl (B.E.); e.g.e.de.vries@umcg.nl (E.G.E.d.V.); 2 Department of Medical Oncology, Erasmus MC Cancer Institute, 3015 GE Rotterdam, The Netherlands; l.angus@erasmusmc.nl (L.A.); a.jager@erasmusmc.nl (A.J.); j.kraan@erasmusmc.nl (J.K.); j.martens@erasmusmc.nl (J.W.M.M.); s.sleijfer@erasmusmc.nl (S.S.); 3 Department of Medical Oncology, Radboud Medical Center, 6500 HK Nijmegen, The Netherlands; evelien.kuip@radboudumc.nl; 4 Department of Medical Oncology, Amsterdam UMC, Vrije Universiteit Amsterdam, Cancer Center Amsterdam, 1081 HV Amsterdam, The Netherlands; c.menke@amsterdamumc.nl; 5 Department of Pathology, University Medical Center Groningen, University of Groningen, 9713 GZ Groningen, The Netherlands; b.van.der.vegt@umcg.nl; 6 Department of Nuclear Medicine and Molecular Imaging, University Medical Center Groningen, University of Groningen, 9713 GZ Groningen, The Netherlands; a.w.j.m.glaudemans@umcg.nl (A.W.J.M.G.); a.h.brouwers@umcg.nl (A.H.B.); 7 Department of Radiology and Nuclear Medicine, Amsterdam UMC, Vrije Universiteit Amsterdam, 1081 HV Amsterdam, The Netherlands; d.oprea-lager@amsterdamumc.nl; 8 Department of Radiology and Nuclear Medicine, Radboud Medical Center, 6500 HK Nijmegen, The Netherlands; woyen@rijnstate.nl; 9 Department of Biomedical Sciences, Humanitas University, 20090 Milan, Italy; 10 Department of Nuclear Medicine, Humanitas Clinical and Research Center, 20089 Milan, Italy; 11 Department of Radiology and Nuclear Medicine, Rijnstate, 6815 AD Arnhem, The Netherlands; 12 Department of Radiology and Nuclear Medicine, Erasmus MC, 3000 KP Rotterdam, The Netherlands; emmeringj@maasstadziekenhuis.nl; 13 Department of Medical Oncology, Netherlands Cancer Institute NKI-AvL, 1066 CX Amsterdam, The Netherlands

**Keywords:** circulating tumor cells, immunohistochemistry, ^18^F-FES-PET, estrogen receptor, metastatic breast cancer, endocrine therapy

## Abstract

**Background/Objectives**: Outcome prediction in patients with estrogen (ER)-positive metastatic breast cancer (MBC) remains challenging. We investigated whether circulating tumor cell (CTC) count adds prognostic value in ER-positive MBC using immunohistochemical (IHC) or 16α-[^18^F]-fluoro-17β-estradiol (FES)-PET imaging. **Methods:** Patients with newly diagnosed non-rapidly progressive MBC receiving first-line endocrine monotherapy, with ER-positive IHC (biopsy) or FES-PET and available CTC count, were included. Associations of CTC count and CTC-ER status based on *ESR1* mRNA expression with progression-free survival (PFS) and overall survival (OS) were analyzed, and the added prognostic value of CTC count (<5 vs. ≥5/7.5 mL) beyond a positive ER result was assessed. **Results**: In patients with ER-positive IHC (*n* = 98) or FES-PET (*n* = 99) out of 106 endocrine-treated patients, ≥5 CTCs were associated with shorter PFS (HR 1.86; *p* = 0.0047 and 1.75; *p* = 0.011) and OS (HR 3.19 and 3.22; both *p* < 0.01), respectively, compared with <5 CTCs. Adding CTC count to ER-positive IHC or FES-PET improved prognostic accuracy for PFS (*p* = 0.006 and 0.012) and OS (both *p* < 0.001). CTC-ER status (*ESR1* RNA) was not associated with outcomes. **Conclusions**: CTC count adds prognostic value to PET- or biopsy-based ER analysis in endocrine-treated MBC.

## 1. Introduction

For patients diagnosed with metastatic breast cancer (MBC), molecular tumor characteristics such as estrogen receptor (ER) status, progesterone receptor (PR) status, and human epidermal growth factor receptor (HER2) status are essential parameters for treatment decision-making. Hence, a tumor biopsy is considered the gold standard for obtaining current information on the receptor status [1]. Molecular imaging, by using 16α-[^18^F]-fluoro-17β-estradiol ([^18^F]-FES) for positron emission tomography combined with computed tomography (PET/CT) [2,3], is a more comprehensive and patient-friendly way to assess whole-body ER status than taking a biopsy [4]. Although both methods accurately identify patients with ER-positive disease and are related to endocrine therapy response and outcome, it is well established that not all patients respond to endocrine therapy [5]. A minimally invasive way to obtain prognostic insight is to determine the circulating tumor cell (CTC) count [6,7]. The CTC count at MBC diagnosis has prognostic value [8,9], but the CTC count is not recommended for routine clinical practice in current guidelines [1,10,11]. A recent review underlines the role of CTCs as a robust prognostic biomarkers in metastatic breast cancer, supporting integrative approaches that combine liquid biopsy with advanced imaging techniques [12].

In addition to CTC count, enriched CTC fractions can be assessed for ER expression by analyzing the level of estrogen receptor 1 (*ESR1*) RNA expression using real-time PCR [7,13].

Currently, it is unknown whether CTC count or CTC-ER status based on estrogen receptor 1 (*ESR1*) RNA expression adds value in predicting outcomes in patients with ER-positive metastatic disease, as defined by positive ER expression by the immunohistochemistry (IHC) of a metastasis or whole-body FES-PET positive result, who are receiving endocrine treatment.

Therefore, in this study, it was evaluated whether CTC count or CTC-ER status based on *ESR1* RNA adds prognostic value to PET- or biopsy-based ER analysis in endocrine-treated patients with MBC.

## 2. Materials and Methods

This study is a sub-study of a Dutch multicenter, prospective, observational cohort study in patients with non-rapidly progressive MBC eligible for first-line systemic therapy (IMPACT-MBC trial; NCT01957332). All patients with MBC, regardless of breast cancer subtype or whether they had bone-only disease, underwent imaging including [^18^F]-fluorodeoxyglucose ([^18^F]-FDG) PET/CT and [^18^F]-FES PET/CT, a metastasis biopsy, and blood sampling as described previously [4]. The relationship between the ER result of metastatic tumor tissue and the FES-PET result has already been reported for the entire cohort [4]. Patients who received first-line endocrine monotherapy (according to standards in the Netherlands), with positive ER expression by IHC performed on a biopsy obtained from a metastatic lesion or a whole-body FES-PET positive result and with available blood CTC assessment, were eligible for the present sub-study. Imaging with a contrast-enhanced CT scan of the thorax/abdomen was performed after 8 weeks and repeated at least 6 and 12 months after treatment initiation. Thereafter, response assessments were performed every 12 months or otherwise at the treating physician’s discretion. Patients with measurable disease were evaluated according to Response Evaluation Criteria in Solid Tumours (RECIST) version 1.1 criteria [14]. For patients with non-measurable disease per RECIST 1.1 criteria, PD was defined as clinical progression meriting the discontinuation of therapy according to the treating clinician, including the substantial worsening of overall complaints or disease-related organ function decline and unequivocal new lesions on CT. Progression-free survival (PFS) was defined as the interval between treatment initiation and PD or death and overall survival (OS) as the interval between treatment initiation and death regardless of cause.

### 2.1. CTC Count and ESR1 Expression

At baseline, 20 mL of blood was drawn, 10 mL in CellSave tubes (Menarini-Silicon Biosystems, Huntingdon Valley, PA, USA) for CTC count and 10 mL in Vacutainer^®^ EDTA tubes (BD, Franklin Lakes, NJ, USA) for CTC *ESR1* RNA expression analysis. Blood tubes were analyzed at the Translational Cancer Genomics Laboratory at Erasmus MC Cancer Institute, Rotterdam, the Netherlands. A detailed description of the CTC count and RNA isolation from CTCs has been reported previously [13,15,16,17]. CTC count was measured in 7.5 mL of CellSave blood within 96 h after collection, using the CellSearch System (Menarini-Silicon Biosystems). Patients were grouped based on CTC count per 7.5 mL of blood into two categories: <5 CTCs and ≥5 CTCs [8,11].

For *ESR1* RNA expression analysis, CTCs were isolated from 7.5 mL EDTA blood within 24 h of collection using the CellSearch system using the CellSearch profile kit (Menarini-Silicon Biosystems). After CTC enrichment, RNA was extracted using the AllPrep DNA/RNA Microkit (Qiagen, Germantown, MD, USA). Subsequently, cDNA was generated and pre-amplified for target genes of interest, including *ESR1* and the listed reference genes, and then quantified by real-time PCR (RT-qPCR) using TaqMan Gene Expression Assays (Applied Biosystems, Carlsbad, CA, USA). For this analysis, samples with ≥5 CTCs were selected and verified for sufficient mRNA signals (average quantification cycle (Cq) < 26.5) based on reference genes glucuronidase beta (*GUSB*)*,* hydroxymethylbilane synthase (*HMBS*) and hypoxanthine phosphoribosyl transferase 1 (*HPRT1*) and sufficient epithelial signals (keratin 19 (*KRT19*)/epithelial cell adhesion molecule (*EPCAM*) average Cq < 26.5), as described previously [8,13]. Based on previous results, CTCs were considered ER-positive if *ESR1* mRNA expression exceeded the predefined threshold of ΔCq ≥ −7.86 relative to the reference genes [13].

### 2.2. Tumor Tissue Analyses

The metastasis biopsy was paraffin-embedded and stained for the standard determination of the histological subtype and ER, PR, and HER2 as previously described [18] and evaluated according to standard procedures by breast pathologists at each center. A central review was performed by an experienced breast pathologist (BvdV), who was blinded to the imaging results.

### 2.3. FES-PET

FES-PET was performed and the results analyzed as described previously [4,19]. All centers were accredited by the European Association of Nuclear Medicine Research Limited (EANM/EARL) [20,21]. For the present analysis, positivity was defined visually (locally and centrally confirmed) when at least one lesion showed uptake above the background or semi-quantitatively if, in at least one lesion, the maximal standardized uptake value (SUV_max_) exceeded the 1.50 threshold in visually doubtful lesions [4].

### 2.4. Statistical Analyses

PFS on first-line endocrine therapy and OS were estimated using the Kaplan–Meier method, with median survival times derived accordingly. Group differences in PFS and OS according to CTC count among patients with ER-positive disease (defined by IHC or FES-PET) and according to CTC-ER status (positive or negative) were evaluated using the log-rank test. Cox proportional hazards regression models were used to assess the association of CTC count and CTC-ER status with outcomes, with the results reported as hazard ratios (HRs) and 95% confidence intervals (CIs).

To assess the added prognostic value of CTC count in patients with ER-positive disease, nested Cox regression models were constructed. A base model was compared with an extended model that additionally included CTC count, within subgroups defined by ER status using IHC or FES-PET. Model fit was evaluated using the −2 log likelihood (−2LL), with lower values indicating better fit. Likelihood ratio tests (LRTs) were used to evaluate whether the inclusion of CTC count significantly improved model fit, thereby indicating added prognostic value.

All statistical tests were two-sided, and *p* values < 0.05 were considered statistically significant. Statistical analyses were performed using IBM SPSS Statistics for Windows (version 28.0) and R for Windows (version 4.4.0).

## 3. Results

### 3.1. Patient Population

**Figure 1:**
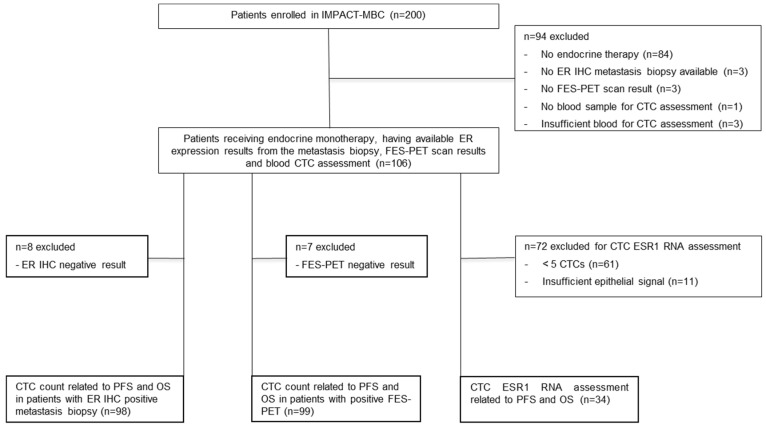
Consort diagram showing patients included in IMPACT-MBC study and eligible for this analysis. Abbreviations: MBC, metastatic breast cancer; ER IHC, estrogen receptor immunohistochemistry; FES-PET, 16α-[^18^F]-fluoro-17β-estradiol positron emission tomography; CTC, circulating tumor cell; *ESR1* RNA, estrogen receptor 1 RNA; PFS, progression-free survival; OS, overall survival.

A total of 200 patients were enrolled between August 2013 and May 2018, with data cut-off in June 2022. One hundred six patients met the eligibility criteria for this sub-study, namely receiving first-line endocrine therapy and having ER expression results from the metastasis biopsy, FES-PET scan results, and CTC assessment. Ninety-eight of them had positive ER expression by IHC, and 99 had a positive FES-PET (Figure 1). CTC counts ranged from 0 to 1377/7.5 mL of blood, with a median of two CTCs (Table 1). In total, 61 patients had < 5 CTCs, whereas 45 had ≥ 5 CTCs. For the ESR1 RNA expression analysis, material from 34 of the 106 patients was evaluable (Figure 1). Progressive disease on first-line endocrine therapy had occurred in 93 patients, whereas 13 remained progression-free. After a median follow-up of 61 months, 43 patients were alive, while 63 patients had died. The median PFS and OS were 18 months and 43 months, respectively.

### 3.2. CTC Count Related to PFS and OS in Patients Treated with Endocrine Therapy

In the total cohort (*n* = 106), the median PFS was 23 versus 14 months for patients with <5 versus ≥ 5 CTCs, respectively, with ≥5 CTCs being associated with shorter PFS (HR 1.85, 95% CI 1.22–2.80; *p* = 0.004, Figure S1). The median OS was 67 months versus 28 months for patients with <5 versus ≥5 CTC count, respectively, with ≥5 CTC count being associated with shorter OS (HR 2.98, 95% CI 1.78–4.97; *p* < 0.001, Figure S1).

The addition of CTC groups improved prognostic accuracy beyond a positive IHC-ER result or FES-PET positive result alone (LRT *p* = 0.004 and <0.001).

### 3.3. Prognostic Value of CTCs in Patients with Positive ER Expression by IHC

**Figure 2:**
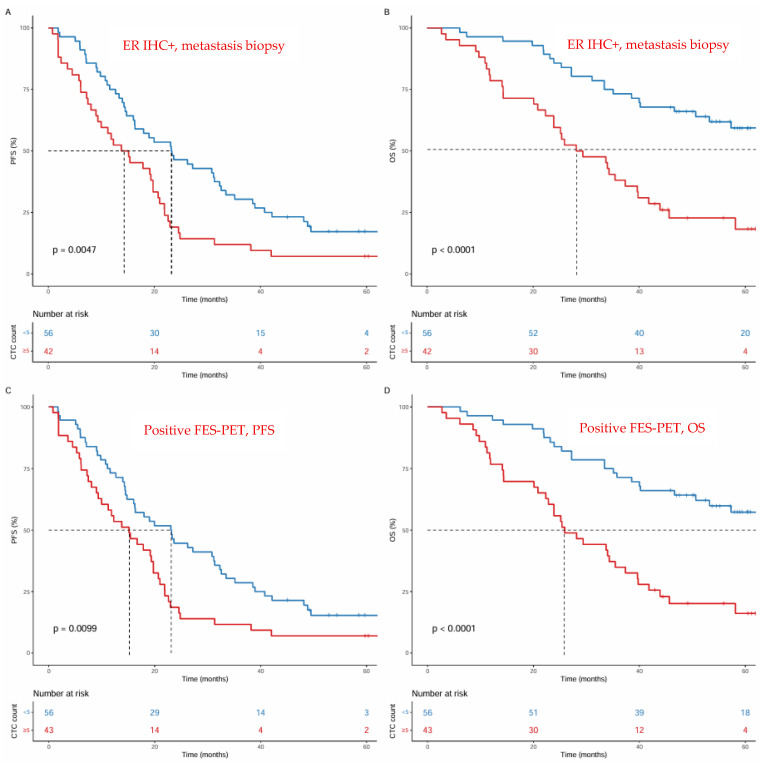
CTC count related to PFS and OS. CTC count < 5: blue line; CTC count ≥ 5: red line. (**A**) PFS in patients with positive ER expression by IHC; (**B**) OS in patients with positive ER expression by IHC. (**C**) PFS in patients with FES-PET positive result; (**D**) OS in patients with FES-PET positive result.

In the group of patients with positive ER expression by IHC (*n* = 98), the median PFS was 23 vs. 14 months for those with <5 versus ≥5 CTCs respectively, with ≥5 CTCs being associated with shorter PFS (HR 1.86, 95% CI 1.20–2.87, *p* = 0.0047; Figure 2A, Table S1). The addition of CTC count improved prognostic accuracy for PFS beyond positive ER expression by IHC alone (−2LL 663.40 vs. 655.81; LRT *p* = 0.006, Table S2).

The median OS was not reached at 29 months for those with <5 versus ≥5 CTCs, with ≥5 CTCs being associated with shorter OS (HR 3.19, 95% CI 1.86–5.48, *p* < 0.001; Figure 2B, Table S1). The addition of CTC count improved prognostic accuracy for OS beyond that of positive ER expression by IHC alone (−2LL 474.24 vs. 456.32; LRT, *p* < 0.001, Table S2).

### 3.4. Prognostic Value of CTCs in Patients with FES-PET Positive Result

In the group of patients with an FES-PET positive result (*n* = 99), the median PFS was 23 vs. 15 months for those with <5 versus ≥ 5 CTCs, respectively, with ≥5 CTCs being associated with shorter PFS (HR 1.75, 95% CI 1.14–2.69, *p* = 0.011; Figure 2C, Table S1). The addition of CTC count improved prognostic accuracy beyond an FES-PET positive result alone (−2LL 677.67 vs. 671.29; LRT *p* = 0.012, Table S2).

The median OS was 67 vs. 26 months for patients with <5 versus ≥ 5 CTCs, respectively, with a ≥5 CTC count being associated with shorter OS (HR 3.22, 95% CI 1.90–5.45, *p* < 0.001; Figure 2D, Table S1). The addition of CTC count improved prognostic accuracy beyond an FES-PET positive result alone (−2LL 497.83 vs. 478.67; LRT *p* = 1.2 × 10^−5^, Table S2).

### 3.5. Prognostic Value of CTC ESR1 RNA Expression

In the group of patients evaluable for CTC *ESR1* RNA expression (*n* = 34), the median PFS was 9 months versus 18 months for patients with CTC ER-positive (*n* = 30) versus CTC ER-negative results (*n* = 4), respectively. The median OS was 25 versus 29 months for patients with CTC ER-positive versus CTC ER-negative results, respectively. No statistically significant differences in PFS or OS were observed between CTC-ER-positive and CTC-ER-negative results (HR 0.72, 95% CI: 0.25–2.08, *p* = 0.55, Figure S2, and HR 0.73, 95% CI: 0.22–2.41, *p* = 0.60, Figure S2, respectively).

## 4. Discussion

In this study, we observed that adding CTC count to the evaluation of ER-positive disease, determined by the IHC of metastatic tumor tissue or an FES-PET positive result, improved prognostic accuracy for PFS and OS in patients with newly diagnosed metastatic breast cancer receiving first-line endocrine monotherapy. This suggests that in patients with hormonally driven disease, CTC count can provide meaningful additional prognostic information. CTC *ESR1* RNA did not provide additional predictive value.

To our knowledge, combining CTC parameters and FES-PET positive results to predict outcomes in MBC has not been investigated previously. This is a unique exploratory study that combines relevant biomarkers, including CTC count and CTC-based *ESR1* RNA expression, with FES-PET imaging in patients treated with endocrine therapy, to examine their relationship with long-term outcomes.

Baseline CTC count has previously been shown to be an independent prognostic marker [22,23,24]. In line with other studies, we also found that patients with ≥5 CTCs had a shorter median PFS and OS than patients with <5 CTCs [12,25,26,27,28]. The implication for treatment choice is currently unclear. Escalating treatment in patients with ≥5 CTCs was shown to improve outcomes in patients with breast cancer of no special type but not in lobular breast cancer [29]. Also, other trials conducted to date have not confirmed the benefit of CTC-informed treatment escalation [30,31]. Currently, the ACT-MBC study is prospectively assessing the impact of CTC count on treatment decisions, response assessment, and prognosis in patients with MBC (NCT05662345). Awaiting these results, the clinical utility of CTC measurements to support treatment decisions or response monitoring has not been proven, and routine CTC testing is not yet part of guidelines [1,10,11]. Nonetheless, our study indicates that adding CTC count to the evaluation of ER-positive disease, defined by IHC or FES-PET, better predicts PFS and OS than a positive ER result defined by IHC or FES-PET alone. Beyond the CTC count at baseline, longitudinal measurements showed that early conversion in the CTC count was associated with outcome, independent of subtype or treatment [9].

In our study, CTC *ESR1* RNA expression was not associated with PFS or OS. This is likely explained by the known phenotypic and molecular heterogeneity in MBC CTCs [32]. Also, our finding may, at least in part, be attributable to the limited number of evaluable patients, as reliable *ESR1* mRNA assessment was only feasible in fewer than one third of this sub-study owing to the requirement of ≥5 CTCs and quality criteria for epithelial markers. Therefore, these findings should be considered exploratory and hypothesis-generating. In clinical practice, the test’s robustness should be improved to provide meaningful results. Diagnostic leukapheresis is a potential approach to increasing CTC yield, which may enable more robust gene expression analysis across a larger patient cohort [33]. Beyond the scope of this analysis, there is potentially informative value in *ESR1* mutation assessment in CtDNA [34]. However, these mutations are still rare in patients with newly diagnosed ER-positive MBC. CtDNA analyses are ongoing in the IMPACT-MBC study.

This study has limitations. Patients with rapidly progressive disease were not included. Although this may have introduced selection bias, for relatively indolent ER-positive disease, the effects are likely to be small. This is a sub-study of a larger imaging study, IMPACT-MBC, and the present data should be considered exploratory. Adding whole-body ER heterogeneity [19] and *ESR* mutation status [35] may be of additional prognostic value. The IMPACT-MBC study was small for a breast cancer study but in fact large for an imaging biomarker trial. In the present study, we performed a dichotomous analysis with a standard cut-off of 5 CTCs/7.5 mL according to guidelines [11], but in light of the wide numerical range we found (0–1377 CTCs/7.5 mL), continuous analysis could potentially provide additional prognostic information. The IMPACT-MBC study was performed with first-line endocrine monotherapy, the standard of care at the time. Although the prognostic impact of our findings is not expected to be affected by adding CDK inhibition, this is currently being investigated in the SONImage trial [36]. The strength of this sub-study is a comprehensive collection of samples within a well-defined study population, including tissue- and molecular imaging-based ER measurements and CTC analysis. All procedures were highly standardized and centralized, with a focus on long-term outcome data. This study included patients with bone-only disease, improving the generalizability of the findings [4,19].

## 5. Conclusions

In patients with ER-positive metastatic breast cancer receiving first-line endocrine therapy, circulating tumor cell count provides independent and additive prognostic information beyond ER assessment by biopsy or FES-PET. These findings support the integration of liquid biopsy with tissue- and imaging-based biomarkers to refine risk stratification in hormonally driven disease. While not yet practice-changing, this multimodal approach represents a promising step toward more individualized prognostic assessment and warrants validation in larger, contemporary cohorts incorporating current standard therapies.

## Figures and Tables

**Table 1:** Patient and disease characteristics.

Number of Patients	*n* = 106
**Sex**	
Female	105 (99)
Male	1 (1)
**Age, years**	
Median (SD), (min, max)	63 (10), (32–83)
**Histology**	
Invasive carcinoma NST	84 (79)
Invasive lobular carcinoma	18 (17)
Other ^ǁ^/unknown *	4 (4)
**ER status (IHC)**	
Positive	98 (92)
Negative	8 (8)
**PR status (IHC)**	
Positive	78 (74)
Negative	27 (25)
Unknown	1 (1)
**HER2 status (IHC)**	
Positive	1 (1)
Negative	104 (98)
IHC unknown	1 (1)
**FES-PET**	
Positive	99 (93)
Negative	7 (7)
**CTC count**	
<5 CTCs	61 (58)
≥5 CTCs	45 (42)
**Median CTC count (range)**	2 (0–1377)
***ESR1* RNA (*n* = 34)**	
positive	30 (88)
negative	4 (12)

Abbreviations: SD, standard deviation; ER, estrogen receptor; PR, progesterone receptor; HER2, human epidermal growth factor 2 receptor; IHC, immunohistochemistry; NST, no special type; FES-PET, 16α-[^18^F]-fluoro-17β-estradiol positron emission tomography; CTCs, circulating tumor cells; *ESR1* RNA, estrogen receptor 1 (*ESR1*) mRNA. ^ǁ^ 1 tubulolobular carcinoma, 1 apocrine carcinoma, and * 2 unknown.

## Data Availability

The original contributions presented in this study are included in the article/Supplementary Material. Further inquiries can be directed to the corresponding author.

## Supplementary Materials

The following supporting information can be downloaded at https://www.mdpi.com/article/10.3390/diagnostics16081197/s1, Figure S1A,B: PFS and OS by CTC *ESR1* RNA status. Figure S2. IMPACT-MBC consortium. Table S1: CTC count related to PFS and OS in patients with positive ER expression by IHC or FES-PET. Table S2: Added prognostic value of circulating tumor cell (CTC) count beyond ER assessment.
