# Supplementary material for: Circulating Tumor Cells Enhance Prognostic Stratification Beyond ER Assessment by Biopsy or FES-PET in Endocrine-Treated Metastatic Breast Cancer
Source: Diagnostics (Basel). 2026 Apr 17;16(8):1197. doi: 10.3390/diagnostics16081197 (PMC13114810; doi:10.3390/diagnostics16081197)
Supplement: Supplementary file 1 [file diagnostics-16-01197-s001.zip › diagnostics-4168001-supplementary.pdf]

These supplementary figures are part of the submission:

Circulating tumor cells add prognostic value to PET- or biopsy-based estrogen receptor analysis in endocrine-treated metastatic breast cancer.

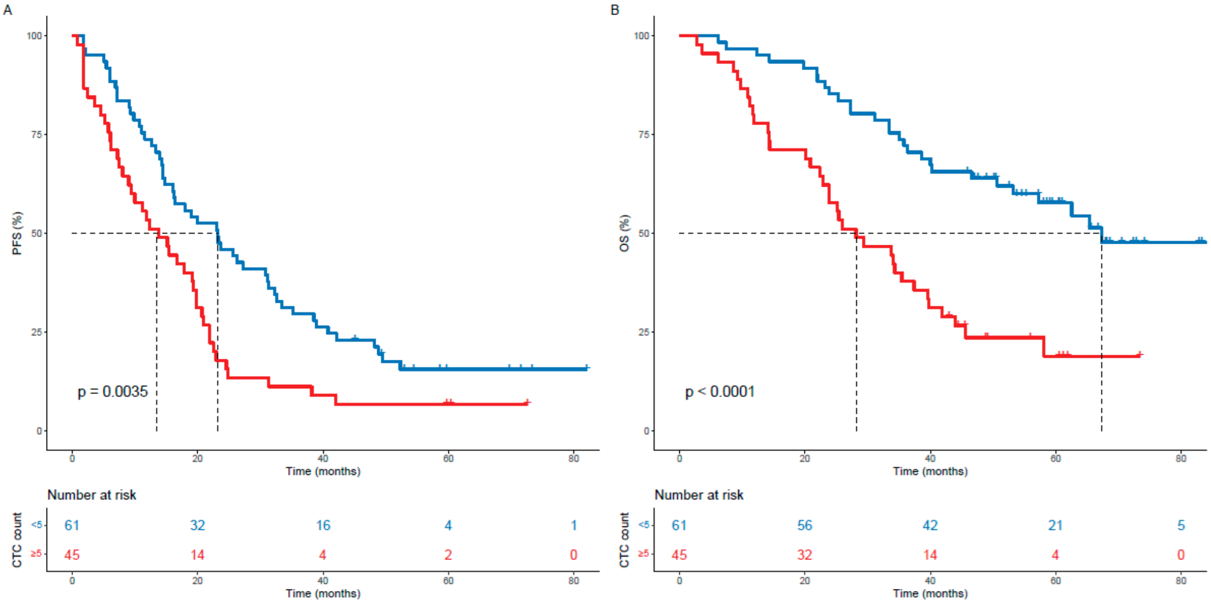

Figure S1. CTC count related to PFS and OS. CTC <5: blue line; CTC ≥5: red line. A: PFS in patients with ER-positive disease defined by IHC of FES-PET results; B: OS in patients with ER-positive disease defined by IHC or FES-PET results.

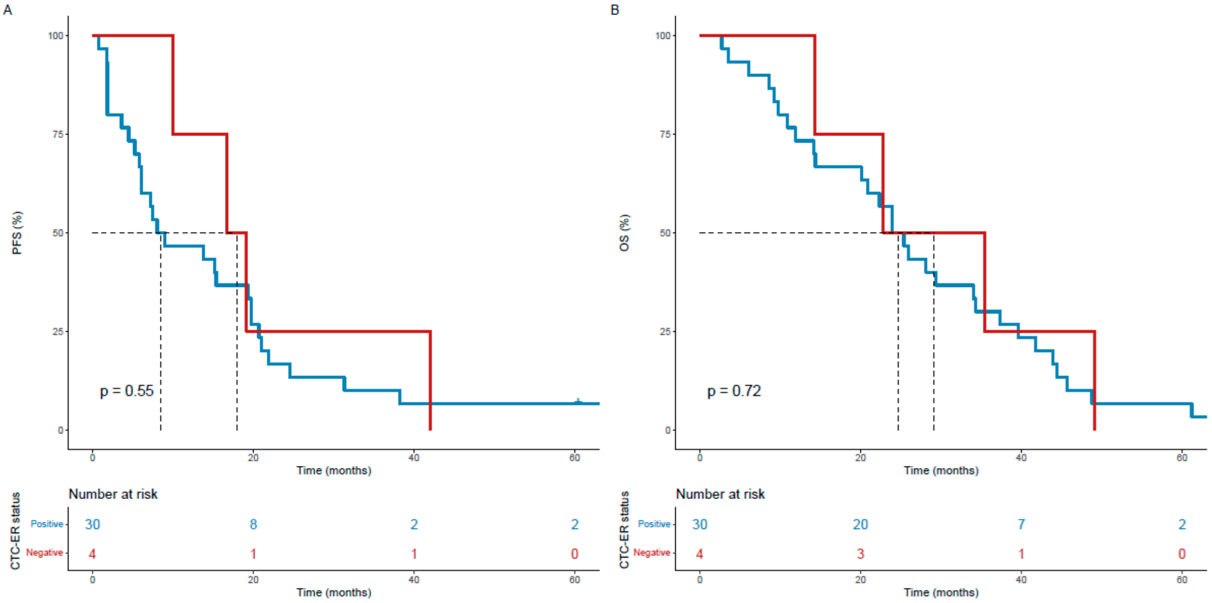

Figure S2. CTC-ER status based on ESR1 mRNA expression related to PFS and OS. CTC-ER-positive: blue line; CTC-ER-negative: red line. A: PFS in patients with CTC-ER-positive or -negative status; B: OS in patients with CTC-ER-positive or -negative status.

Table S1: CTC count related to PFS and OS in patients with positive ER expression by IHC or FES-PET.

		CTC <5	CTC ≥5	Hazard ratio	p-value
ER IHC+	PFS (months)	23	14	1.86	0.0047
	OS (months)	Not reached	29	3.19	< 0.001
FES-PET-positive	PFS (months)	23	15	1.75	0.011
	OS (months)	67	26	3.22	< 0.001

Table S2: Added prognostic value of circulating tumor cell (CTC) count beyond ER assessment.

**ER assessment**	**Outcome**	**Variables included**	**−2 Log Likelihood**	**LRT p-value**
**IHC**	PFS	Positive ER expression by IHC	663.40	—
**IHC**	PFS	Positive ER expression by IHC + CTC count	655.81	0.006
**IHC**	OS	Positive ER expression by IHC	474.24	—
**IHC**	OS	Positive ER expression by IHC + CTC count	456.32	<0.001
**FES-PET**	PFS	FES-PET positive result	677.67	—
**FES-PET**	PFS	FES-PET positive result + CTC count	671.29	0.012
**FES-PET**	OS	FES-PET positive result	497.83	—
**FES-PET**	OS	FES-PET positive result + CTC count	478.67	1.2×10^{-5}
